# Folate Intake and Breast Cancer Risk: A Systematic Review and Meta‐Analysis

**DOI:** 10.1002/mnfr.70354

**Published:** 2025-12-23

**Authors:** Mariana Reis Eleotério, Larissa Souza Ferreira, Francilene Maria Azevedo, Aline Carare Candido, Sylvia do Carmo Castro Franceschini, Eliana Carla Gomes de Souza

**Affiliations:** ^1^ Department of Nutrition and Health Federal University of Viçosa – Campus Viçosa Minas Gerais Brazil

**Keywords:** breast cancer, DNA methylation, risk

## Abstract

This study aimed to evaluate the relationship between folate intake and breast cancer risk in adult women.

A blinded paired‐matched search was conducted in Embase, PubMed/Medline, and Web of Science. Cohort and case‐control studies assessing folate intake or supplementation and breast cancer risk were included. The search yielded 377 studies. After exclusion of duplicates and reading of the titles and abstracts, 13 were selected for full‐text reading, and 11 met the eligibility criteria. Most studies (*n* = 8) found a direct association between folate intake and reduced breast cancer risk, with one study observing a U‐shaped relationship between dietary folate intake and breast cancer risk. Conversely, three studies found no association with breast cancer risk. Some studies report inconsistencies in the literature. A meta‐analysis comparing mean folate intake between case and control groups revealed no statistical significance. Some studies have indicated a possible association between folate consumption and the risk of breast cancer, however the meta‐analysis of this study did not confirm this relationship. Results were inconsistent, and the meta‐analysis indicated no significant effect.

## Graphical abstract

Although folate is involved in the one‐carbon cycle and DNA methylation, there are still inconsistencies between articles that investigated the interrelationship between dietary or supplemental folate consumption and the risk of breast cancer, and the meta‐analysis did not indicate statistical significance, indicating no effect.

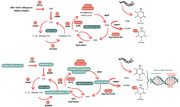

**Abbreviations**
BRCA: breast cancer gene
CH3: methyl group
DHF: dihydrofolate
DNA: deoxyribonucleic acid
DNMT: DNA methyltransferase
SAM: S‐adenosylmethionine

## 1 Introduction

Cancer is a term that encompasses more than 100 malignant diseases characterized by the disorderly growth of cells, which can divide rapidly, form tumors, and invade adjacent tissues or distant organs [1]. In 2020, breast cancer was the most commonly diagnosed cancer worldwide, with over 2.26 million new cases. In 2022, despite an estimated increase to over 2.31 million new cases, breast cancer became the second most common cancer. It is the most common cause of cancer death in women and the fourth most common cause of cancer death overall [2]. Globally, annual breast cancer deaths are estimated to reach nearly 1 million by 2030, and over 70% of these deaths will occur in low‐ and middle‐income countries [3].

Breast cancer is the most prevalent among women and has a higher risk related to age, early menarche, physical inactivity, excess weight, and unbalanced dietary intake [4]. Epigenetic modifications are also considered risk factors. It is worth noting that epigenetics involves studying non‐coding heritable alterations in the DNA sequence, which play an important role in gene expression. Epigenetic alterations include DNA methylation, microRNA activity, and histone modification, with DNA methylation being the most widely studied form of epigenetic modification [5].

One of the risk factors for cancer is aberrant genetic and epigenetic events, such as aberrant DNA methylation. This biological process involves adding methyl groups to cytosine residues, a biological process that depends on the availability of methyl groups and, consequently, on the function of methyl donors and acceptors. In this sense, micronutrients such as folate, choline, betaine, vitamin B12, and other B vitamins contribute to DNA methylation as methyl donors and cofactors. Folate maintains genomic stability by regulating DNA biosynthesis, repair, and methylation, as well as the biosynthesis of nucleotides, amino acids, neurotransmitters, and S‐adenosylmethionine (SAM). SAM is the primary methyl group donor, controlling gene transcription and protein expression through its ability to methylate cytosine in the DNA molecule. Thus, folate status may correlate with aberrant DNA methylation and the risk of developing breast cancer [6].

However, the role of folate in carcinogenesis is complex, as it may play a dual role in the development of cancer, with either a protective or a risk effect, depending on the amount ingested. A meta‐analysis found a preventive effect with a daily intake of between 320 and 400 µg of folate. However, daily folate intake levels greater than 400 µg were associated with an increased risk of breast cancer [7, 8]. It is worth noting that the estimated average folate recommendation from the Dietary Reference Intakes (2000) is 400 µg per day for adults [9].

There are limitations regarding the effects of folate on breast cancer, as they are considered complex and may vary depending on the context. Studies have shown contradictory results: at low concentrations, folate may have protective effects, whereas at high doses, it may contribute to disease progression. This phenomenon hinders the establishment of clear recommendations on dietary intake and folate supplementation in patients with breast cancer.

Given this, the relationship between folate and breast cancer risk remains uncertain, with no consensus on whether its role is protective or harmful in the context of the disease. Therefore, the aim of this review was to assess the interrelationship between folate intake and the risk of breast cancer in adult women.

## 2 Methodology

### 2.1 Study Protocol and Registration

The systematic review was performed according to the Preferred Reporting Items for Systematic Reviews and Meta Analysis (PRISMA) protocol and was registered in the International Prospective Register of Systematic Reviews (PROSPERO) under protocol CRD42024562384.

### 2.2 Research Sources

The PICOS strategy was adopted to define the research question and search strategy, with the population (P) being adult women, the intervention (I) being folate intake, the comparison (C) being different levels of intake, the outcome (O) being breast cancer and the study design (S) being cohort or case control. Thus, the guiding question was: “Is folate intake associated with the risk of developing breast cancer?”. The search was conducted by blinded peers between 04/04/2024 and 30/07/2024 in the Embase, PubMed/Medline and Web of Science databases. The descriptors and Boolean operators used were: “DNA Methylation” AND “breast cancer risk” OR “breast neoplasms risk” OR “breast tumors risk” AND “folate” OR “folic acid” OR “methyl donor nutrients” OR “one carbon cycle nutrients.” The search key was adapted for each database, as shown in Table S1.

### 2.3 Study Selection Criteria

Original cohort or case‐control studies, without restriction on date or study location, in all languages, that associated folate intake or supplementation with the risk of developing breast cancer were included. Experimental studies, reviews, editorials, abstracts, commentaries, reports, pilot study protocols, and those that evaluated only plasma folate levels were excluded.

### 2.4 Study Selection and Data Extraction

The studies identified in the databases were entered into the Rayyan software for selection [10]. Initially, duplicates were excluded. Next, the titles and abstracts were analyzed independently by two authors (M.R.E. and L.S.F.). Subsequently, conflicts were discussed, and a third author was consulted (F.M.A.) in the event of disagreement. Thus, potentially eligible studies were identified according to the inclusion and exclusion criteria. The selection followed the steps of reading titles and abstracts and then reading them in full. The following data were extracted from the studies: author and year of publication, design, study location, study duration, type of supplementation, folate dosage, and main results.

### 2.5 Risk of Bias Assessment

The risk of bias was analyzed using the Joanna Briggs Institute's Critical Analysis Tool for Cohort and Case‐Control Studies. The risk of bias was considered low when ≥ 70% of the responses were affirmative (“yes”), moderate if between 50, and 69% and high when ≤ 49% [11]. However, this assessment was not used as an inclusion criterion.

### 2.6 Statistical Analysis

A meta‐analysis was conducted to compare the mean folate intake between case (cancer) and control (cancer‐free) groups. The sample sizes, mean, and standard deviation of folate intake for both groups were extracted for the meta‐analysis. For studies in which the mean and standard deviation were not presented, the medians and interquartile range were converted to mean and standard deviation using the method of Wan et al. [12], and for studies that presented minimum and maximum medians, the method of Hozo et al. [13] was applied for estimation.

Statistical analysis was performed in RStudio software, version 4.4.1, using the metacont function of the meta package [14]. The groups were compared using the standardized mean difference (SMD), with their respective 95% confidence intervals (95% CI). The influence graph generated from the InfluenceAnalysis function of the dmetar package was used to identify studies that contributed to greater heterogeneity. At this stage, we identified that one study contributed significantly to data heterogeneity, which its exclusion reduced to low heterogeneity [17] (Figure S1). Therefore, the study was removed from the analysis. The fixed effects model was used, since the heterogeneity observed between the studies was low (*I* ^2^ < 50%). Forest and funnel plots were generated to visualize the results and assess publication bias, respectively.

## 3 Results

### 3.1 Study Selection

**FIGURE 1:**
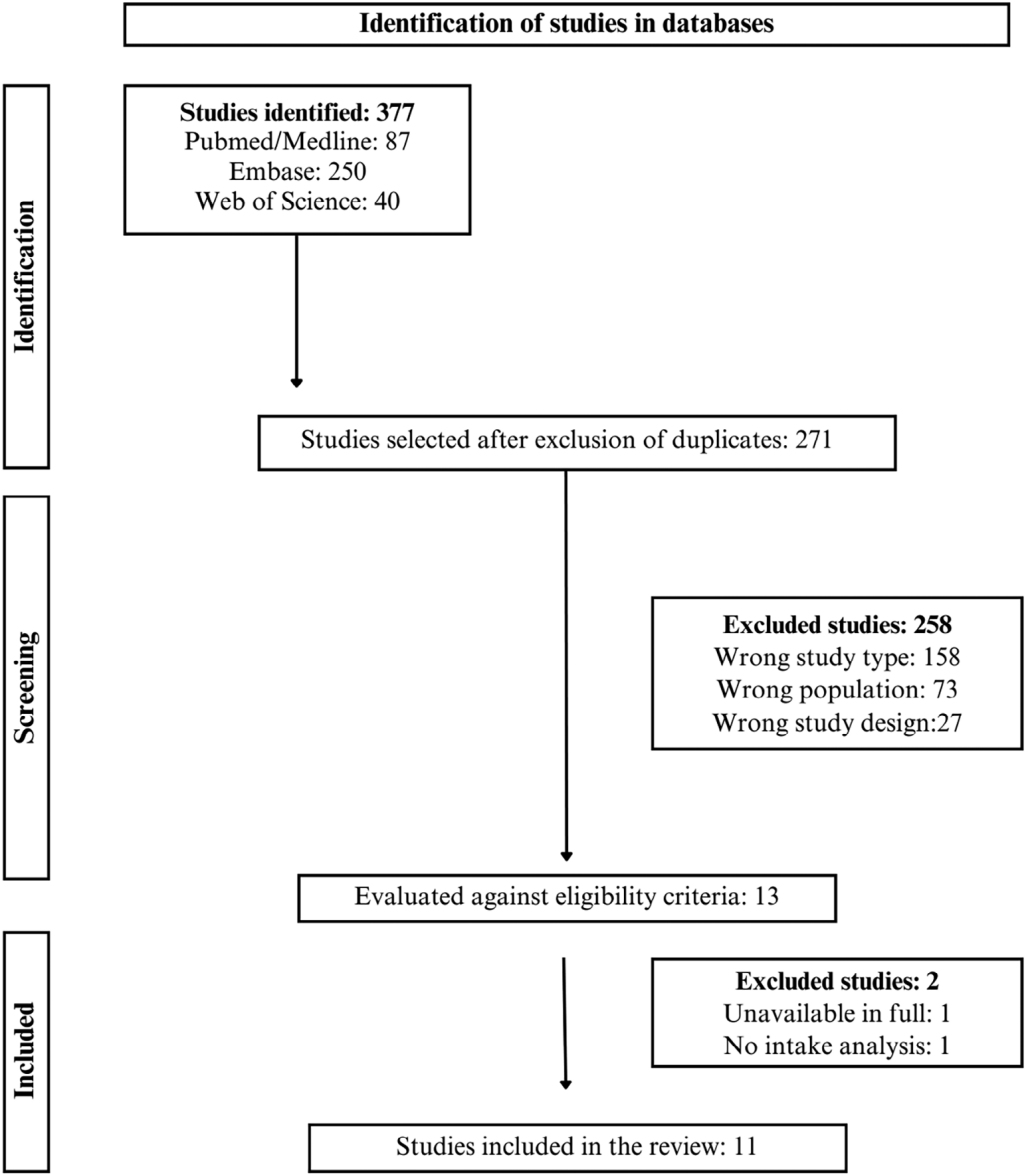
Flowchart of the study selection process. PRISMA [15]

The database search yielded 377 studies, totaling 271 after duplicates were removed. After reading the titles and abstracts, 13 were selected for full‐text reading and 11 met the eligibility criteria (Figure 1).

#### 3.1.1 Summary of Results

Of the 11 studies selected to compose the systematic review, 5 were case‐control studies [16, 17] and 6 were cohort [18, 19, 20, 21, 22, 23, 24, 25, 26, 27] as shown in Table S2. In all case‐control studies, the case groups were composed exclusively of women diagnosed with breast cancer.

To assess folate intake, 10 studies applied food frequency questionnaires, 2 of which included questions about the use of supplements and one study applied only the questionnaire about the use of supplements.

Most studies (*n* = 8) found a direct association between folate intake and reduced breast cancer risk, with one study observing a U‐shaped relationship between dietary folate intake and breast cancer risk [27]. Furthermore, one of the studies observed an association between folate intake and a lower risk of breast cancer in women who regularly consumed alcohol [19]. On the other hand, three studies observed no association with disease risk [14, 16, 19]. The studies were carried out in different countries, with two conducted in the United States, one in France, one in Sweden, one in Canada, one in Denmark, one in China, one in Germany, one in Italy, and one was developed in several countries, namely: Denmark, France, Germany, Italy, Norway, Spain, Sweden, the Netherlands, and the United Kingdom.

The age of the women in the studies ranged from 25 to 80 years. The sample of the selected cohort studies totaled 577 487 women who were followed for 4.7 to 17.4 years. In the case‐control studies, there were a total of 2248 cases of breast cancer and 2389 controls.

All studies investigating the use of folate supplements considered both the consumption of multivitamins containing the micronutrient and the isolated use of folic acid [19, 24]. Shrubsole et al. [17] reported that 6.2% of the women evaluated regularly used vitamin B supplements or multivitamins. Larsson, Bergkvist, Wolk [24] observed that 24.5% of the participants reported the use of multivitamins, while only 1.0% used exclusively folic acid supplements. In turn, Kim et al. [20] found that total folic acid intake from multivitamins and other supplements varied widely among participants, with values ranging from 0 to 1239.17 µg/day.

#### 3.1.2 Risk of Bias Assessment

**FIGURE 2:**
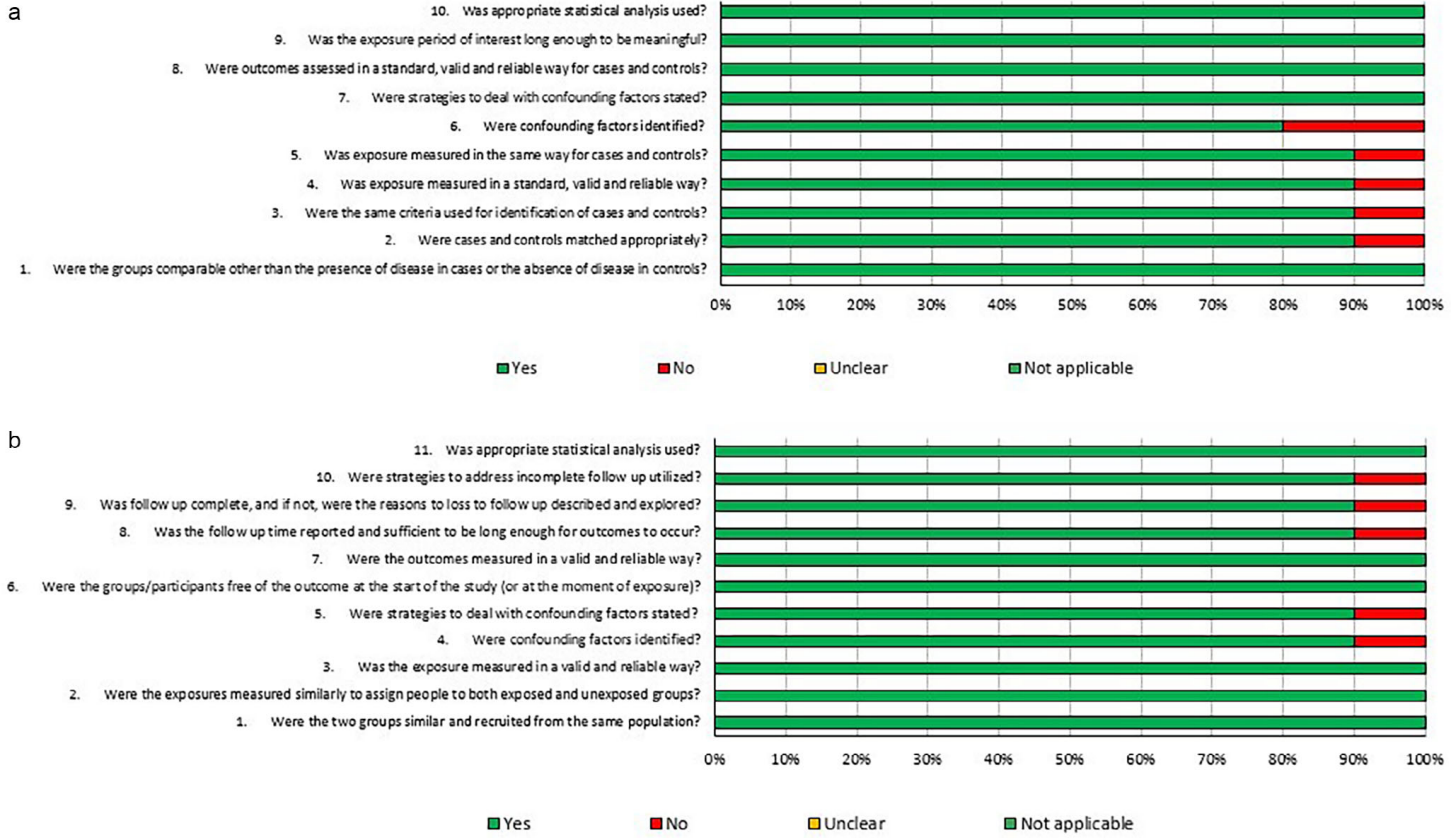
(a) Risk of bias graph for case‐control studies. (b) Risk of bias graph for cohort studies.

The risk of bias assessment was performed according to the study design, with positive responses above 70% in all studies of both designs (Figure 2). Regarding cohort studies, two did not identify confounding factors, and one did not declare strategies to deal with these confounding factors. Furthermore, one study did not match cases and controls adequately, and another did not clarify whether the groups were comparable. Regarding case‐control studies, one study did not use strategies to address incomplete follow‐up, one did not report the reasons for sample losses, and one did not identify confounding factors and or declare strategies to deal with these confounding factors

#### 3.1.3 Meta‐Analysis

**FIGURE 3:**
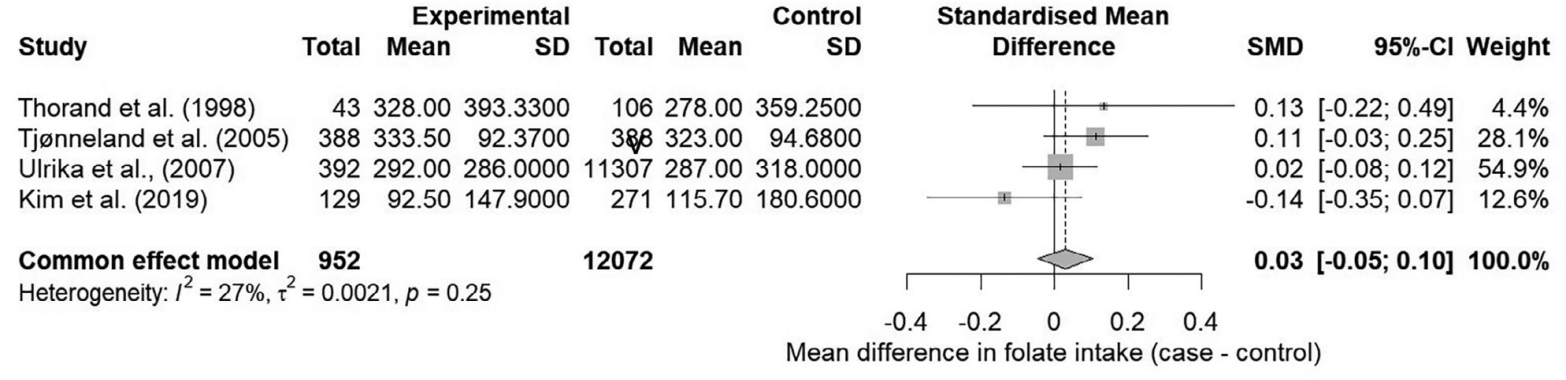
Forest plot of meta‐analysis.

Four studies were included in the meta‐analysis with a total sample of 12 072 individuals. The pooled effect size (SMD), represented by the mean difference in folate intake between cases and controls, was 0.03 (CI: −0.05; 0.10), therefore not significant. Heterogeneity was low (*I* ^2^ = 27%), which indicates the possibility of using the fixed effect (Figure 3 ).

**FIGURE 4:**
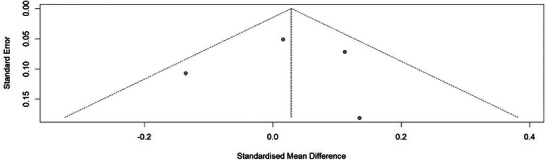
Funnel plot of publication bias assessment.

In the analysis of publication bias, the studies show good symmetry, which indicates the absence of publication bias (Figure 4).

## 4 Discussion

Most studies have found a direct association between folate intake and reduced risk of breast cancer, and one study found a U‐shaped relationship between dietary folate intake and breast cancer risk.

**FIGURE 5:**
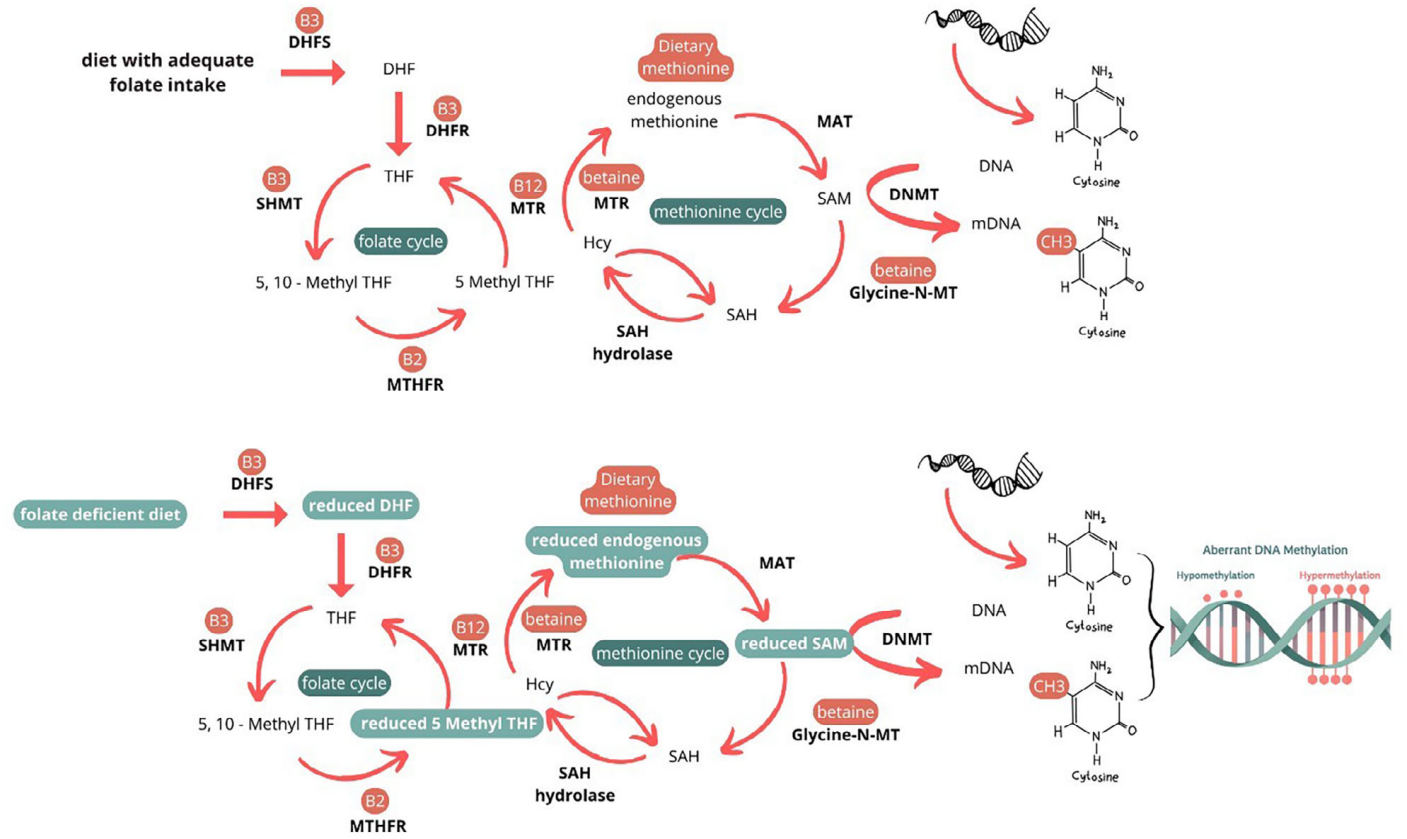
Alterations in the one‐carbon cycle in the face of a folate‐deficient diet. *Source*: Author, 2025.

One of the mechanisms that associates folate with cancer is aberrant DNA methylation, since it is associated with the availability of these nutrients in a cellular process known as “one‐carbon metabolism” (Figure 5). It provides methyl groups for biological methylation, an essential epigenetic mechanism for regulating genetic transcription, where a methyl group (CH3) is transferred from S‐adenosyl‐L‐methionine to carbon 5 of a cytosine. One‐carbon metabolism begins by converting dietary folate to dihydrofolate (DHF), the pathway's main element. In addition, adequate S‐adenosyl‐L‐methionine (SAM) synthesis is essential for the process to occur biologically, which acts as a DNA methyltransferase (DNMT) cofactor and a universal methyl donor for DNA methylation. Faced with folate deficiency, there is a change in SAM concentration, which favors aberrant methylation, either by hypomethylation or hypermethylation of DNA [6].

The studies analyzed indicate that adequate folate intake reduces the risk of breast cancer due to its participation in the one‐carbon cycle and in the maintenance of genomic stability, as it plays an important role in DNA synthesis and methylation reactions [17, 18, 19, 20, 22, 23, 25, 26].

This result aligns with a meta‐analysis conducted by Chen et al. [28], which identified an inverse relationship between daily dietary folate intake and breast cancer risk in case‐control studies. The analysis showed that an increase of 100 µg per day in folate consumption was associated with a reduction of approximately 9% in the risk of the disease, compared with women whose intake was less than 130.5 µg per day.

Furthermore, the authors of the previous meta‐analysis observed that higher folate intake could reduce the risk of breast cancer in women with higher alcohol intake [28]. This finding is in line with some studies analyzed in this review, where Tjønneland et al. [19] observed that adequate folate intake may attenuate the risk of breast cancer associated with high alcohol consumption. Zhang et al. [21] found that higher total folate intake or multivitamin use was also associated with a lower risk of breast cancer among women who regularly consumed alcohol.

In the cohort study conducted by Puyvelde et al. [27], no evidence of an association between individual dietary folate intakes and breast cancer risk was found. However, when considering only dietary intake, the authors suggested a U‐shaped relationship in the general population, indicating that both low and high intake levels could be associated with increased risk. In contrast, intermediate levels would appear more protective. This pattern was also observed in the meta‐analysis by Chen et al. [28], which identified a similar relationship in prospective cohort studies. In this analysis, women with daily dietary folate intakes between 153 and 400 µg had a lower risk of developing breast cancer compared to those who consumed less than 153 µg/day. On the other hand, intakes greater than 400 µg/day did not confer significant additional protection. A similar result was reported by Lajous et al. [22] who found an inverse association between folate intake and breast cancer risk, with a median intake of 393 µg/day among participants. It is important to highlight that, according to the Dietary Reference Intakes (2000), the tolerable upper intake level (UL) of folate for adults is 1000 µg/day, a value far from the intakes observed in the studies. In contrast, the reported intakes are close to the estimated average requirement (EAR) of 400 µg/day, also established by the IOM [9].

Given this, in the cohort of Maruti, Ulrich, White [25] of supplement users, the association between folate intake across quartiles of intake was examined. The results suggest that high folate intakes (1272 µg/day) over approximately 10 years reduced the risk of breast cancer, since those who consumed high doses of total folate had a 22% lower risk of breast cancer than women in the lowest intake category. Furthermore, Ulrika et al. [23] found a reduced incidence of invasive breast cancer in the highest quintile of dietary folate intake, total folate intake and dietary folate equivalents, including supplements and dietary folate equivalents. In contrast, Kim et al. [20] indicated that intake of 8.56–89.29 µg/day of folic acid supplements decreased the risk of breast cancer, while intake above 89.29 µg /day showed no association.

Furthermore, it is worth noting that several countries have adopted mandatory fortification of flours and cereals with folic acid, which may influence the nutrient consumption data. Some studies suggest that this measure may lead to underestimating the real folic acid intake, especially when the contribution of fortified foods is not considered. Furthermore, in populations with higher intakes, the data may reflect the post‐fortification period [20, 25].

It is worth noting that some studies indicate that high doses of folate, through a fortified diet and supplements, are a concern, because folate from synthetic sources (folic acid) is more bioavailable than that from food. This supplementation may favor the progression of the disease if pre‐neoplastic lesions are present [25].

According to the latest report from the World Cancer Research Fund International, which evaluated the last decade of research on cancer prevention and the links between diet, nutrition, physical activity, and cancer, the evidence on the association between folate intake and breast cancer remains limited and inconclusive. Furthermore, regarding supplementation, it clarifies that there is no substantial evidence that supplements can reduce the risk of the disease [28].

Among the six studies that investigated the association with postmenopause, four observed an inverse relationship between folate intake and breast cancer risk [19, 22, 25]. Kim et al. [20] observed a decreased risk of breast cancer among women with BRCA1 mutation who used folic acid supplements compared with women who never used them. Folate deficiency in cells can result in increased DNA strand breaks, chromosomal instability, and chromosomal repair, which, together with insufficient BRCA protein due to inherited mutation, can propagate and promote neoplastic transformation.

Furthermore, three selected studies associated folate intake with a lower cancer risk according to subtypes. However, they obtained different results, with the studies citing the subtypes ER+, ER−, PR+, ER+PR+, ER+/PR−, and HER2 [24, 25, 26].

In addition, three of the selected studies, all case‐control studies, found no association [16, 18, 21]. In the case‐control study by Thorand et al. [16], categories were created to represent diets with low, intermediate, and high availability of the methyl group, namely folate and methionine. A diet high in folate and methionine was inversely associated with breast cancer, both for total folate and folate equivalents. However, when dietary intake was adjusted for total energy intake, the inverse association disappeared, and a diet high in folate and methionine showed a moderate positive association, but it was not statistically significant.

In the cohort of Zhang et al. [21], neither total folate intake nor intake derived from food alone was associated with breast cancer risk. However, higher total folate intake or multivitamin use was associated with a lower risk of breast cancer among women who regularly consumed alcohol.

In the case‐control study by Zhu et al. [18], the analysis of dietary folate intake suggested an increased risk with lower folate intake for cases with methylated genes, reduced risk for cases with unmethylated genes, and intermediate risk for cases with unknown methylation status. Although this pattern was consistent with the study hypothesis, it did not reach statistical significance.

These results corroborate some cohort studies that also found no association between folate intake and breast cancer risk [29, 30, 31, 32]. It is worth noting that the studies indicate that the presence of confounding factors may bias the results, in addition to the variation in cutoff points for folate intake categories. Furthermore, they indicate that it is unlikely that folate deficiency in the human diet is as pronounced as in animal studies. Furthermore, they indicate that folate can potentially promote tumor cell growth, and high blood folate levels may be associated with an increased risk of breast cancer [18, 21].

Throughout this review, studies with divergent and inconclusive results were identified. Evidence is inconsistent and does not confirm a protective role of folate against breast cancer. The meta‐analysis reinforces this uncertainty, since it did not show a significant effect, indicating that the available data are still insufficient and that further studies are necessary. Our result corroborates the meta‐analysis of prospective studies by Zhang et al. [21], which also did not observe any effects of folate intake on the incidence of breast cancer.

As a limitation of this review, there may be possible confounding factors, such as the fortification of foods with folic acid in some countries and the lack of complete information about the dosage of folic acid consumed, which may bias the results. In addition, the cutoff points for the categories of folate intake differed significantly between studies.

The review's potential was to include a broad population range representing different countries. Furthermore, this systematic review is considered unprecedented in its proposed format, allowing for investigating the interrelationship between folate intake and the risk of breast cancer. It is also worth highlighting the meta‐analysis, which strengthens the findings by allowing for the quantitative synthesis of the data and increasing the robustness of the evidence gathered.

## 5 Final Considerations

Although some studies have suggested a possible association between folate intake and breast cancer risk, the meta‐analysis of this study did not confirm this relationship, indicating an absence of a significant effect. Further well‐designed, large‐scale studies are needed to clarify the relationship between folate.

## Funding

This study was funded by the Coordenação de Aperfeiçoamento de Pessoal de Nível Superior (CAPES), under process number 88887.900533/2023‐00.

## Conflicts of Interest

The authors declare no conflicts of interest.

## Supporting information




**Supporting File 1**: mnfr70354‐sup‐0001‐SupMat.pdf.

## Data Availability

Data sharing not applicable to this article as no datasets were generated or analyzed during the current study.
