# Supplementary material for: Folate Intake and Breast Cancer Risk: A Systematic Review and Meta‐Analysis
Source: Mol Nutr Food Res. 2025 Dec 23;70(1):e70354. doi: 10.1002/mnfr.70354 (PMC12728386; doi:10.1002/mnfr.70354)

Supplementary table 1

Databases	Search strategy
**Pubmed**	((*DNA Methylation)* AND (*breast cancer risk* OR *breast neoplasms risk* OR *breast tumors risk)*" AND (*folate* OR *folic acid* OR *methyl donor nutrients* OR *one carbon cycle nutrient)).*
**Web of Science**	((*DNA Methylation)* AND (*breast cancer risk* OR *breast neoplasms risk* OR *breast tumors risk)*" AND (*folate* OR *folic acid* OR *methyl donor nutrients* OR *one carbon cycle nutrient)).*
**Embase**	''*DNA Methylation*' AND '*breast cancer risk'* OR '*breast neoplasms risk*' OR '*breast tumors risk*' AND 'f*olate'* OR '*folic acid*' OR '*methyl donor nutrients*' OR '*one carbon cycle nutrients*'.

Supplementary table 2

Authors, year, and place	Sample	Design	Intake assessment	Folate dosage	Time	Main results
Thorand et al. (1998), Germany	Postmenopausal women, with 43 cases and 106 controls.	Case-control. Consumption of fruits, vegetables, folic acid, methionine, cysteine, and alcohol was assessed.	Online FFQ, containing 201 items.	Median of cases 21µg/day3 and controls 203µg/day.	-	No solid evidence that a high intake of folic acid reduces the risk of breast cancer.
Zhang et al. (1999), USA	88,818 women	Prospective cohort. Mailed questionnaires about medical history and lifestyle were completed and nutrient intakes were calculated.	FFQ	Risk reduction with at least 600 µg/day.	16 years	Total folate intake was not associated with overall breast cancer risk. However, ↑ total folate intake or multivitamin use was associated with a ↓ breast cancer risk among

						women who regularly consumed alcohol.
Shrubsole et al. (2001), China	Women, 1321 cases and 1382 controls, who never drank alcohol regularly or used vitamin supplements	Case-control. Anthropometrics and dietary habits were assessed to analyze whether folate is associated with reduced risk of cancers.	76-item FFQ, developed and tested for use in this population	Average intake of 294µg/day ± 172.	-	Dietary folate intake was inversely associated with breast cancer risk. This inverse association was strongest among women whose usual diets contained a ↑ level of methionine, vitamin B2, vitamin B6, and vitamin B12.
Zhu et al. (2003), USA	African-American women, with 304 cases with breast cancer and 305 controls 305 women without cancer.	Case control. Dietary methyl content was assessed by methionine, folate and alcohol intake.	Questionnaire that includes foods that account for at least 90% of total U.S. intake of each of the 18 major nutrients FFQ with 192 items.	-	-	No association was observed between a methyl-deficient diet and breast cancer.

Tjønneland et al. (2005), Denmkark	Postmenopausal adult women, with 388 cases and 388 controls.	Case-control nested within a cohort. Information on dietary intake and use of supplements (oral) was collected and it was analyzed whether the increased risk of breast cancer associated with alcohol intake can be reduced by adequate folate intake.	FFQ with 192 items.	Cases: 358 µg; controls: 348 µg.	4.7 years	Adequate intake (300 mg/day) of folate may ⬇ the risk of breast cancer associated with high alcohol intake.
Lajous et al. (2006), France	62,739 postmenopausal women.	Prospective cohort. Dietary issues were assessed with regular questionnaires assessing folate, alcohol, vitamin B2, and B12 intake.	FFQ of 208 food items, beverages and recipes	Median intake of 393 µg/day.	9 years	There was an inverse association between folate intake and breast cancer risk, which was greater among women who reported high vitamin B12 intakes.

Larsson, Bergkvist, and Wolk (2008), Sweden	36,664 healthy women.	Cohort. Dietary intake was assessed to analyze folate consumption and assess whether there is an association with the risk of ER and PR breast cancer.	FFQ with 67 and 96 foods and supplements of folic acid, B vitamins and multivitamins.	Mean dietary intake: 234 ± 50 µg.	17.4 years	An inverse association was observed between folate intake and the risk of ER+/PR- breast cancer; however no association was observed with total breast cancer or ER+/PR+ or ER-/PR- tumors.
Maruti, Ulrich, and White (2009), USA	35,023 postmenopausal women.	Cohort. Researches evaluated the consumption of folate, methionine, riboflavin and vitamins B6 and B12 and supplements with the incidence of breast cancer.	FFQ with 120 items and information collected on intake of multivitamins and vitamin supplements.	Median of 345 µg/day in the lowest quartile and 1272 µg/day in the highest quartile.	5 years	Women who consumed ≥1272 µg/d of total folate for >10 years had a 22% lower risk of breast cancer than women in the lowest category of folate intake. Folate intake was protective for women with ER− breast cancer.
Kim et al. (2019), Canada	Adult women, 129 cases and 271 controls.	Case control with women. Evaluated the use of folic acid, vitamin B6 and vitamin	Food supplement use questionnaire.	Ranged from 0 to 1239.17 mcg/day.	-	Intake of 8.56 to 89.29 mcg/day of folic acid ⬇ risk of breast cancer. Intake above 89.29 mcg/day did not

		B12 supplements (oral) among BRCA mutation carriers.				demonstrate protection. Use of any supplement containing folic acid ⬇ risk of breast cancer among BRCA1 mutation carriers, but not among women with BRCA2 mutation.
Puyvelde et al. (2021), Denmark, France, Germany, Italy, Norway, Spain, Sweden, the Netherlands, and the United Kingdom	318,686 pre- and postmenopausal women.	Prospective cohort. To assess whether methyl group donors, including folate, choline, betaine, and methionine, may influence breast cancer risk.	FFQ for each country or center.	Up to 350 µg/day	14.1 years	No evidence was found for an association between individual dietary folate intakes and breast cancer risk. However, for dietary folate intake, a U-shaped relationship with breast cancer risk was suggested in the general population.
Ulrika et al., (2007), Sweden	11,699 postmenopausal women	Prospective cohort. To assess whether folate intake is	Modified dietary history based on interview and FFQ with 168 items.	Average of 238 µg/day.		⬇ in the hazard ratios for breast cancer incidence in the highest quintile of dietary folate intake, total

	associated with breast cancer incidence.		intake, and dietary folate equivalents compared with the lowest quintile of intake.

Legend: FFQ: Food Frequency Questionnaire; ER: breast tumor estrogen receptor status; PR: breast tumor progesterone receptor; ⬇: lower/decrease; ⬆: higher/increase.

Supplementary figure 1

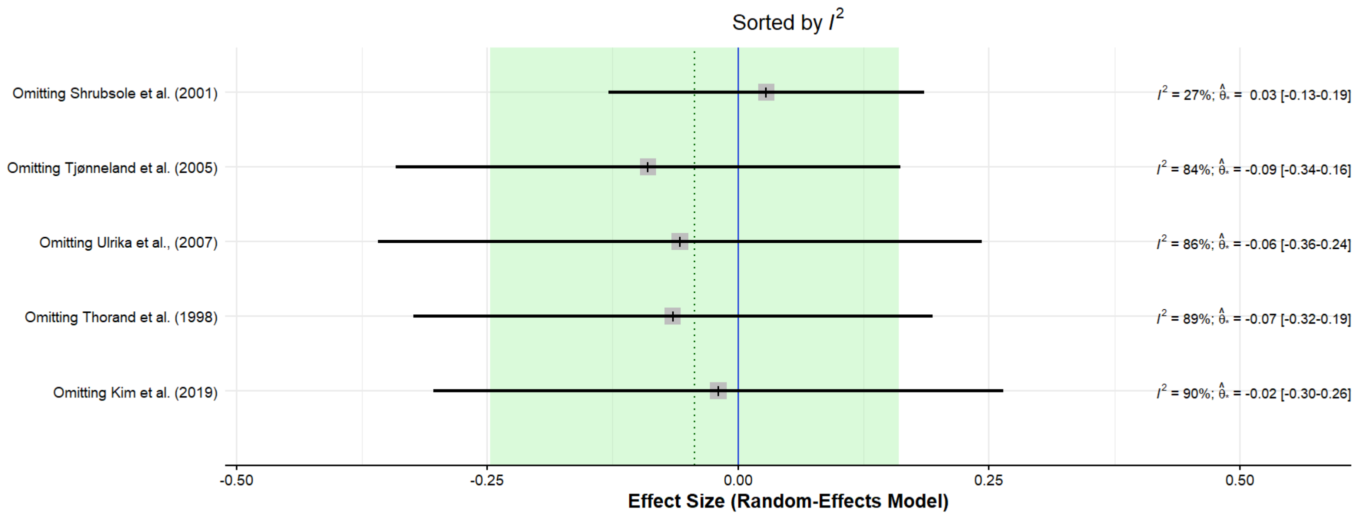

Supplement: Supplementary file 1 — Supporting File 1: mnfr70354‐sup‐0001‐SupMat.pdf. [file MNFR-70-e70354-s001.pdf]
